# A Chitinase from *Aeromonas veronii* CD3 with the Potential to Control Myxozoan Disease

**DOI:** 10.1371/journal.pone.0029091

**Published:** 2011-12-19

**Authors:** Yuchun Liu, Zhigang Zhou, Wei Miao, Yuting Zhang, Yanan Cao, Suxu He, Dongqing Bai, Bin Yao

**Affiliations:** 1 Key Laboratory for Feed Biotechnology of the Ministry of Agriculture, Feed Research Institute, Chinese Academy of Agricultural Sciences, Beijing, People's Republic of China; 2 Key Laboratory of Aquatic Biodiversity and Conservation, Institute of Hydrobiology, Chinese Academy of Sciences, Wuhan, People's Republic of China; 3 Tianjin Key Laboratory of Aqua-Ecology and Aquaculture, Fisheries Science Department, Tianjin Agricultural University, Tianjin, People's Republic of China; Technion-Israel Institute of Technology Haifa 32000 Israel, Israel

## Abstract

**Background:**

The class Myxosporea encompasses about 2,400 species, most of which are parasites of fish and cause serious damage in aquaculture. Due to the concerns about food safety issues and limited knowledge of Myxozoa life cycle and fish immune system, no chemicals, antibiotics or immune modulators are available to control myxozoa infection. Therefore, little can be done once Myxozoa establishment has occurred.

**Methodology/Principal Findings:**

In this paper we isolated *Aeromonas veronii* CD3 with significant myxospore shell valve-degrading ability from pond sediment. A 3,057-bp full-length chitinase gene was consequently cloned, and the corresponding mature, recombinant chitinase (ChiCD3) produced by *Escherichia coli* had substantial chitinase activity. The deduced sequence of ChiCD3 contained one catalytic domain, two chitin-binding domains, and one putative signal peptide. ChiCD3 had an optimal activity at 50°C and pH 6.0, and retained more than 50% of its optimal activity under warm water aquaculture conditions (∼30°C and pH ∼7.0). After incubation with ChiCD3, 38.0±4.8% of the myxospores had damaged shell valves, whereas myxospores incubated with commercially available chitinases remained intact.

**Conclusion/Significance:**

This study reveals a new strategy to control myxozoan disease. ChiCD3 that has capacity to damage the shell valve of myxospores can be supplemented into fish feed and used to control Myxozoa-induced diseases specifically.

## Introduction

The phylum Myxozoa is an entirely endoparasitic group of organisms, especially the fish, which produces complex multi- cellular spores [1]. Myxozoa infection often occurs in fish at gill tissues, fins, kidneys, stomach, swimbladders or serosa of the internal organs and causes pathological changes by forming huge plasmodia [2]. As Myxozoa is responsible for important economic losses among fisheries and aquaculture industries, there are high interests in studying the method to control them. However, the complexity of Myxoan provides a major challenge for the research and control. Class Myxozoa encompasses approximately 2,400 species, of which more than 2,180 species have been characterized, but only about 40 life cycles (less than 2%) are known [1]. Furthermore, little is known about the biology, physiology and parasite-host interactions of most Myxozoa species.

To control myxozoan disease, there are numerous studies focused on the route of immunization, such as selection of myxozoan-resistant fish species and development of vaccines. Even innate resistance of certain fish species against Myxozoan has been reported, but the underlying mechanisms have not been elucidated in most cases [3]. Furthermore, there are inter-specific and intra-specific differences in susceptibility of certain fish species against Myxosporea [3]. For instance, some salmonid species are resistant to *Ceratomyxa shasta* [4] and rainbow trout strains vary in their susceptibility to *Myxobolus cerebralis* [5]. Furthermore, as many myxozoan species elicit little or no host immune responses, it is difficult to characterize the fish immune system and its regulation during infection which is crucial for the development of vaccines [3]. And there is no substantial progress in the exploitation of vaccines. Therefore, a new strategy to control myxozoa infection is urgent.

Generally myxospores consist of 2 to 12 shell valves that typically contain substantial amounts of chitin [6], [7] and are strongly resistant to chemical/antibiotic drugs. These drugs are also limited in their applicability due to the concerns about food safety issues [8]. Chitin is a polymer of *N*-acetyl-d-glucosamine (GlcNAc) and is an important element of fungal cell walls. Chitinases have been widely used to control fungal diseases [9]–[11]. Because chitin is the main component of myxospore shell valves, chitinase as the alternative biological agent to control myxozoan disease would be of great interest. This study is to present a novel chitinase with the ability to degrade the shell valves of myxospores.

## Results

### Myxozoa identification

The mature myxospores were separated from plasmodia in the gut of a common carp (*Cyprinus carpio*). The spores were pyriform with slight tapering anterior and round posterior ends and measured 18.5±0.5 µm (n = 30) long and 8.9±0.6 µm (n = 30) wide. A single flask-shaped polar capsule lied close to the apex of the spores. Even the mean spore length and width of each species was not identical between species, but the morphological and the scanning light microscopy revealed a smooth spore surface of the sample, which is consistent with that of *Thelohanellus* [12]. The 18S rDNA gene fragment was amplified from the genomic DNA of myxospores and it had 99% identity to that of *Thelohanellus kitauei* (accession no. GQ396677) and 96% to *T. hovorkai* (accession no. DQ231155). *T*. *kitauei* is a typical gut parasite of *Thelohanellus*. This site-dependent character differs *T*. *kitauei* from other species without morphological identification [13]. Thus the myxozoa was identified to belong to *T. kitauei*, Myxobolidae, Myxosporea.

### Isolation and identification of strains with shell valve-degrading ability

By using chitinase-screening medium, four bacterial strains were isolated from the sediment of a fish pond which had Myxozoa-induced disease outbreak. Using shell valve of spores as carbon source in culture medium, the reducing sugars (110 µg ml^–1^) were detected in the culture supernatant of strain CD3. Comparison of its 16S rDNA sequence with those in GenBank and a phylogenetic analysis classified strain CD3 as *Aeromonas veronii* (99% identical to *A. veronii* CYJ202, accession no. FJ940848). Then the strain CD3 was deposited into the China General Microbiological Culture Collection Center under registration number CGMCC 3169.

### Chitinase gene cloning and sequence analysis

**Figure 1:**
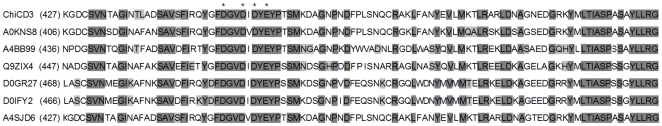
Amino acid sequence alignment of ChiCD3 with the chitinases by CLUSTAL. A0KNS8 from *Aeromonas hydrophila* subsp. *hydrophila* ATCC 7966, A4BB99 from *Reinekea* sp. MED297, Q9ZIX4 from *Pseudoalteromonas* sp. S9, D0GR27 from *Vibrio mimicus* MB-451, D0IFY2 from *Vibrio* sp. RC586 and A4SJD6 from *Aeromona salmonicida* subsp. *salmonicida* A449. The positions of the known catalytic residues in the chitinases are indicated by asterisks. Identical residues are boxed in black and conserved residues are boxed in gray.

**Figure 2:**
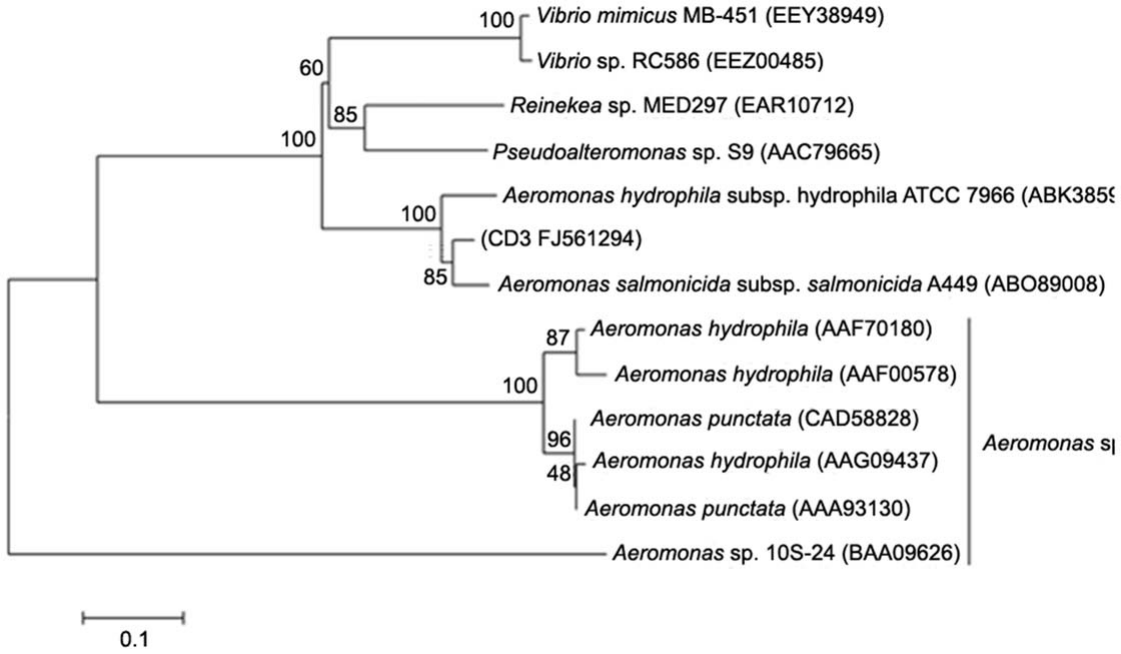
Phylogenetic analysis of ChiCD3 with known chitinases based on the amino acid sequences. The reference sequences were retrieved from GenBank. Bootstrap values (n = 1,000 replicates) are reported as percentages. The scale bar gives the number of changes per amino acid position. The position of ChiCD3 is highlighted in bold type. GenBank accession numbers are given for each species.

The chitinase gene *chiCD3* was isolated from strain CD3 using the degenerate primers and the method of TAIL-PCR. Full-length *chiCD3* contained 3,057 bp and encoded a protein of 1,018 amino acids, including a putative signal peptide of 33 residues, two carbohydrate-binding modules (CBMs) (residues Tyr37–Ala88 and Tyr974–Ala1,016) and one catalytic domain (residues Arg318–Asp768) of glycoside hydrolase family 18. The mature protein had a theoretical molecular mass of 110 kDa. The deduced amino acid sequence of ChiCD3 was 90% identical to those of *Aeromonas salmonicida* subsp. *salmonicida* A449 and *Aeromonas hydrophila* subsp. *hydrophila* ATCC 7966, 53% to the *Vibrio mimicus* MB-451 chitodextrinase precursor and 52% to the *Vibrio* sp. RC586 chitodextrinase precursor. Multiple sequence alignment (Figure 1) indicated that ChiCD3 contained the consensus sequence (DXXDXDXE) of family 18 chitinases [14]. Phylogenetic tree was built using the sequences of family 18 chitinases from *Aeromonas* spp., GenBank and SWISSPROT databases and those found in the literature indicates that ChiCD3 was most closely related to the family 18 chitinases (Figure 2).

### Expression, purification, and characterization of *ChiCD3*

The gene *chiCD3* encoding the mature protein was expressed in *E. coli* BL21 (DE3). After induction with 0.1 mM IPTG (final concentration) at 15°C for 24 h, substantial chitinase activity (2.56 U ml^–1^) was found in the cell lysate supernatant, and no chitinase activity was detected in the lysate supernatant of an uninduced culture or a culture that harbored an empty pET-30a(+).

**Figure 3:**
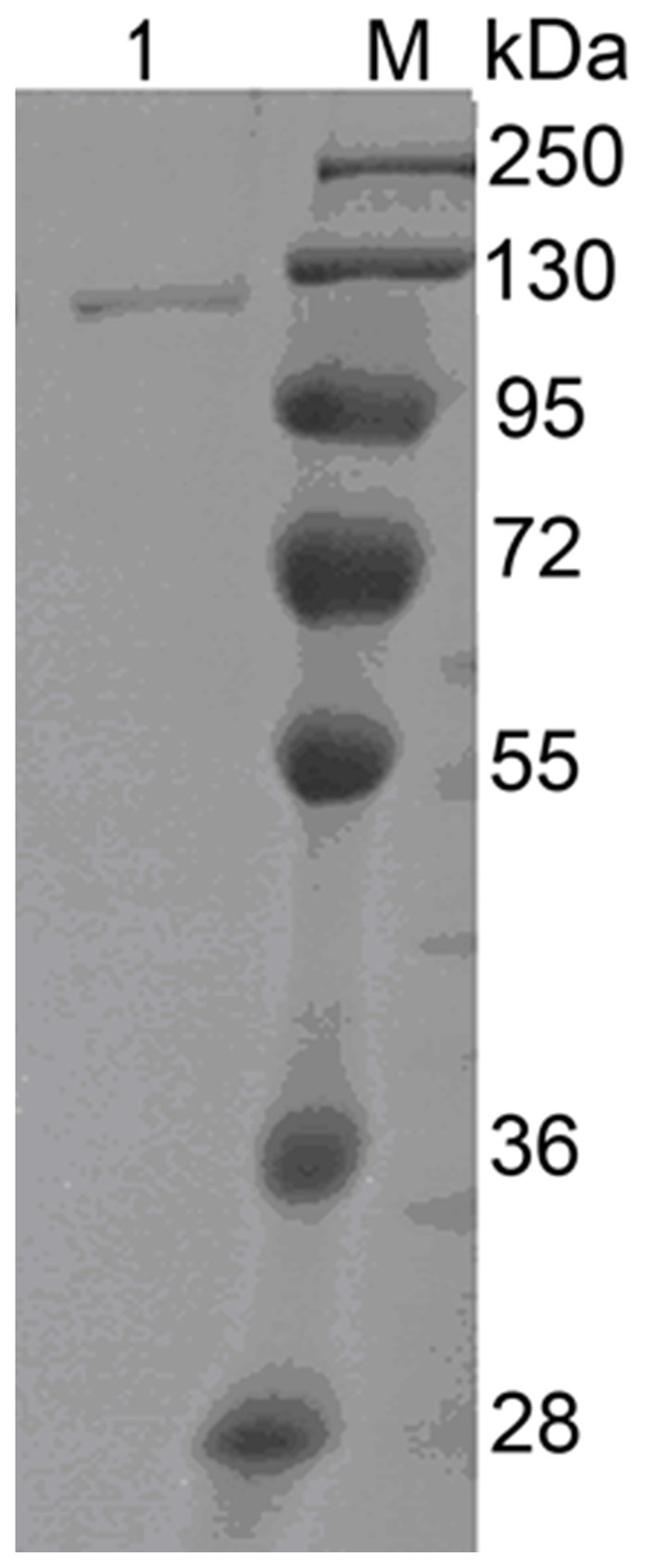
SDS–PAGE gel of purified ChiCD3. Lanes: M, standard protein size markers (kDa); 1, ChiCD3 after purification by Ni^2+^–NTA chelating affinity chromatography. The gel was stained with Coomassie blue.

ChiCD3 was purified to electrophoretic homogeneity by Ni^2+^-NTA metal-chelating affinity chromatography. The purified protein migrated as a single band on SDS–PAGE (Figure 3). The sequences of three internal peptides TLISVGGWADTR, LFANYEVLMK, and EIGGGAVPMWHAK that were recovered from the tryptic digest and identified by tandem mass spectroscopy were also found in the deduced amino acid sequence, which confirmed that the purified protein was indeed that expressed in *E. coli*.

### Enzymatic properties of ChiCD3

**Figure 4:**
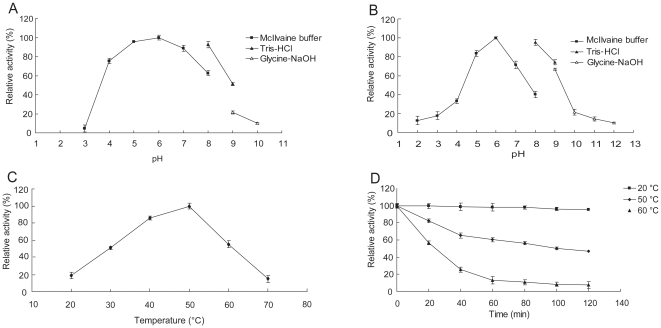
Characterization of the environmental properties that affect the activity of ChiCD3. (A) Effect of pH on activity at 50°C. (B) Effect of pH on the stability of ChiCD3. (C) Effect of temperature on activity at pH 6.0. (D) Effect of temperature on the stability of ChiCD3.

Using colloidal chitin as substrate, ChiCD3 showed the highest activity at pH 6.0, 50°C and more than 80% of the maximum activity was retained at pH 7.0 (Figure 4A). The enzyme retained more than 60% of its maximum activity after 2-h incubation in buffers with different pH values (pH 5.0 to 9.0) at 37°C (Figure 4B). The optimal temperature for ChiCD3 was 50°C at pH 6.0 (Figure 4C). ChiCD3 was stable at 20°C for at least 2 h, whereas at 60°C its activity decreased rapidly (Figure 4D). Under warm water aquaculture conditions (∼30°C and pH∼7.0), the enzyme retained more than 50% of its optimal activity.

### Substrate specificity and kinetic parameters

ChiCD3 had the greatest activity against colloidal chitin (9.2±0.3 U mg^–1^) followed by barley β-glucan (5.3±0.8 U mg^–1^) and carboxymethyl cellulose (4.6±1.2 U mg^–1^). It had no ability to degrade birchwood xylan or locust bean gum. The kinetic constants *K_m_* and *V_max_* for the hydrolysis of colloidal chitin were 7.12 mg ml^–1^ and 19.11 µmol min^–1^, respectively.

### Degradation of shell valve with chitinases

**Figure 5:**
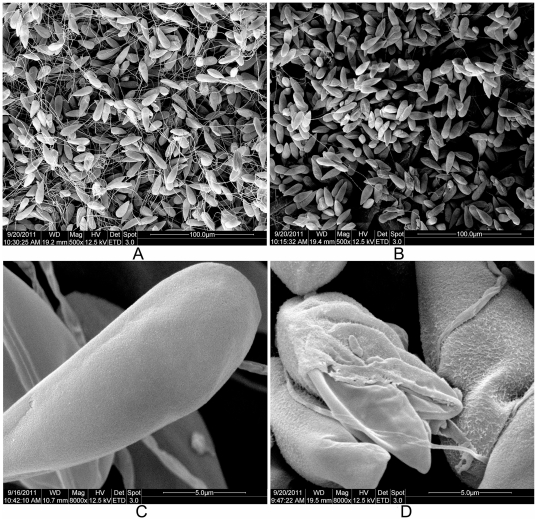
Scanning electron microscope images of myxospores. (A, C) Spores without chitinase treatment. (B, D) Spores treated with recombinant ChiCD3.

The myxospores were treated with 19 U of chitinases from *Streptomyces griseus* and *Serratia marcescens* and the ChiCD3, respectively. Substantial amount of reducing sugar (2.63±0.8 mg l^–1^; Table 1) and damaged shell valves (38.00±4.84% of the total; Table 1, Figure 5) were only detected in the suspension treated with ChiCD3. When treated the myxospores with different concentrations of ChiCD3 (Table 1), obvious dose-response relationship was observed (*P*<0.05).

**Table 1:** The effect of ChiCD3 on myxospores^1^.

Chitinase activity (U)	1.9	19	190
Reducing sugar (mg ml^–1^)	1.06±0.12^a^	2.63±0.80^a^	12.76±1.70^b^
Percentage of damaged myxospores (%)	21.65±6.21^a^	38.00±4.84^ab^	55.69±3.80^b^

1 Data (mean ± SD) in the same row sharing a common superscript are not significantly different (Duncan's multiple-range test, *P*>0.05).

## Discussion

As the complexity of Myxozoa, it is difficult to control the Myxozoan infection by current available methods. *Thelohanellus* spp. are generally histozoic and highly host-specific parasites [15]. As a well-known disease in Southeast Asian countries [12] and less well studied myxosporean group, the genus *Thelohanellus* should be paid more attention to be studied. Of them, *T. kitauei* is the causative agent of the intestinal giant cystic disease of carp, which is a well-known disease in China [16]. Serious infection by *T. hovorkai* causes destruction of capillaries in carp [17]. Because mature *Thelohanellus* spores can remain viable in sediment for at least 5 months [18], the economic damage caused by *Thelohanellus* infection to the aquaculture industry is substantial and increasing. However, the period in sediment also provides a chance to control *Thelohanellus* by damaging the spores. Furthermore, this treatment can be carried out either in aquaculture stage or in the interval stage, and is easy for application by comparison with other researches concerning the parasite life-cycle, parasite-host interaction and fish immune system.

In this study, we isolated an *A. veronii* strain, named as CD3, with extracellular chitinase activity, from pond sediment. To overcome the initial host defense barriers and provide nutrients for cell proliferation, *Aeromona*s strains secrete varieties of proteolytic enzymes [19]. These enzymes might be capable of disrupting myxospores. By using a selective medium with myxospores as the carbon source, *A. veronii* CD3 showed ability to utilize myxospores and its chitinase ChiCD3 produced in *E. coli* also had the ability to damage myxospores by degrading their shell valves.

Comparison of the ChiCD3 catalytic site with that of other bacterial chitinases identified the conserved consensus sequence F_453_DGVDIDYEY_462_, which is essential for chitinase activity [14] and is considered to be the “Prosite signature” for the family 18 members [20] (Figure 1). Chitinases isolated from fungi, plants and bacteria have potent antifungal activity against a wide variety of pathogens [21]. Similar to the fungal cell wall which protects the organism against a hostile environment and relays signals for invasion and infection [21], chitin has also been detected in a variety of members of the phylum Myxozoa [6], [7]. By cleaving the chitin polymers in shell valves, the chitinase will weaken shell valves and render spores osmotically sensitive. Therefore it can be applied to control Myxozoa by degrading the shell valves of Myxospores or to improve the efficacy of other remedies. And integrated control is one of reasonable approach for the complexity of Myxozoan.

In comparison with homologs that have been characterized, ChiCD3 was more active at a less basic pH and at a lower temperature, i.e., pH 8.0–9.0 and 50°C for the former [22] and pH 7.0 and 30°C for ChiCD3. Because ChiCD3 had catalytic activity at a pH and temperature similar to those commonly found for aquaculture environments. In China, the Myxozoa-induced diseases usually outbreak from May to September when the water temperature is 25–38°C [23]. At this temperature range, ChiCD3 retained 30–80% of the maximal activity. Analysis of substrate specificity indicated the enzyme also had activity towards cellulose and β-glucan. There are many reports concerning glycans and lectins (carbohydrate-binding molecules) on the spores surface which form a mutual recognition system and enable parasitic organisms to attach themselves to the host cells [24], [25]. Even the glycans and lectins are not the major components in the shell for Myxospore protection, they are important for specific adhesion and infection of parasites [26]. Therefore the activity towards cellulose and glucan also helps to prevent Myxozoa infection by damaging the interaction. Thus ChiCD3 might provide a possibility for application in aquaculture as a deterrent against Myxozoa infection.

The life cycle of *Thelohanellus* is unknown and it cannot be cultured *in vitro* [3]. Without these experimental data, no transmission model is available. Therefore an attack experiment to assess myxospore vitality with ChiCD3 treatment is very important. In further studies we will focus on the efficacy assessment under application conditions by adding to rearing water. In conclusion, this study provides a new strategy to control myxozoan disease. And it can be easily applied to treat the spores in sediment by comparison with other researches concerning the parasite life-cycle, parasite-host interaction and fish immune system. A novel chitinase ChiCD3 was isolated, which can be used to control the myxozoan by rendering spores osmotically sensitive, degrading the shell valves or preventing parasitic attach to the host cells.

## Materials and Methods

### Ethics

The locations for sample collection are not privately-owned or protected in any way and no specific permits are required for the described field studies and sample collection. Also the field studies does not involve endangered or protected species.

### Myxozoa collection and identification

A Myxozoa-infected common carp that had plasmodia in its gut was taken from an aquaculture pond in Wuqing, Tianjin, China in July 2007. The pond was ∼1.5 m deep, had a temperature of 30°C and a pH of 7.5–8.0 and their surface areas were ∼100 acres. Plasmodia that were filled with mature spores were separated from the fish and ruptured with a needle. Myxospores were washed with sterile 0.7% (w/v) NaCl_(aq)_, collected by centrifugation at 4°C, 500 × *g* for 5 min, and stored in 1.5 ml microfuge tubes at 4°C. Measurements of spore and capsule dimensions were performed using a Leica DM2500 microscope attached with a digital camera (Leica DFC 420, Leica Microsystems, Mannheim, Germany) and ImageProPlus v6 (Media Cybernetics) image analysis system.

Genomic DNA was extracted and purified [27] with some modifications. Briefly, spores were suspended in 1 ml of 500 mM NaCl, 50 mM Tris-HCl (pH 8.0), 50 mM EDTA, 1% sodium dodecyl sulfate (SDS) and 400 µg ml^–1^ proteinase K, and homogenized at the maximum speed for 5 min on a mini Beadbeater in the presence of 600 mg of sterile zirconia beads (100 mg of 0.1 mm and 500 mg of 0.5 mm beads). After incubation at 50°C for 30 min, the crude DNA was centrifuged at 16,000 × *g*, 4°C for 5 min and purified as in [27]. 18S rDNA was PCR amplified using the primers myxoF and myxoR specific for family Myxobolidae [28]. The PCR program was 94°C for 5 min; 35 cycles at 94°C for 30 s, 50° C for 30 s, and 72°C for 1 min; and a terminal extension at 72°C for 5 min. The PCR product was agarose gel purified, ligated to a pGEM-T vector (Promega, USA), transformed into *E. coli* DH5 (TaKaRa, Japan) and sequenced by Sangon (China). The Myxozoa taxon was identified by comparison of its 18S rDNA sequence with those in GenBank.

### Isolation of bacterial strains with shell valve-degrading ability

The chitinase screening medium was prepared as previously described [29] with some modifications. One gram of crab-shell chitin (Sigma, USA) was dissolved in concentrated HCl (100 ml) at 4°C for 24 h. The mixture was filtered through glass wool into ethanol (2 l) at 4°C with rapid stirring. The colloidal chitin pellets were collected by centrifugation at 10,000 × *g* for 20 min, washed repeatedly with sterile H_2_O until a neutral pH was reached, lyophilized and stored at –20°C. The screening medium contained (per liter) 1 g colloidal chitin (the sole carbon source), 3 g KH_2_PO_4_, 2 g K_2_HPO_4_, 1 g NH_4_Cl, 200 mg MgSO_4_·7H_2_O, 10 mg CaCl_2_·2H_2_O and 2% agar, pH 7.0.

Sediment sample was collected from an aquaculture pond in Tianjin, China that had been the site of a Myxozoa-induced disease outbreak and suspended in sterile 0.7% (w/v) NaCl_(aq)_. The suspension was spread onto chitinase screening agar plates and incubated at 25°C for 3 d. Colonies were streaked onto the same medium to obtain purity.

The stock myxospores were suspended in 1 ml of 70% ethanol and centrifuged at 1,000 × *g* for 5 min. This step was repeated twice. Then the spores were suspended in sterile 0.7% (w/v) NaCl_(aq)_ and supplemented into chitinase screening medium to replace colloidal chitin as the inducer and the only carbon source [30]. To screen bacterial strains with shell valve-degrading ability, pure cultures were grown in the screening medium for 5 d. Reducing sugars and chitinase activity in the culture supernatants were assayed as described below. The strain, named CD3, having the greatest chitinase activity was selected for further study.

Genomic DNA of strain CD3 was extracted using DNA Isolation Kit (TIANGEN, China). For identification, 16S rRNA gene fragment was PCR amplified using the primers 27F (5′-AGAGTTTGATCMTGGCTCAG-3′) and 1492R (5′-CGGYTACCTTGTTACGACTT-3′) and sequenced by Sangon.

### Chitinase gene cloning and sequence analysis

A primer set (CHI-F: 5′-GGIGGITGGACIYTIWSICC-3′ and CHI-R: 5′-ATGCAITAYGAYTTYCAYGG-3′) was designed to include the nucleotide sequences for the two conserved motifs GGWTLSD and SVGAWAD of chitinases belonging to family 18 (http://www.cazy.org/GH18.html) and used to touchdown-PCR amplify the *chiCD3* core region. The touchdown-PCR program was: 94°C for 5 min; 5 cycles at 94°C for 30 s, 55°C for 30 s that was decreased by 1°C each cycle, and 72°C for 30 s; 30 cycles at 94°C for 30 s, 50°C for 30 s, and 72°C for 30 s; and a final extension at 72°C for 8 min. The PCR product was purified and ligated into a pGEM-T Easy vector for sequencing. To obtain the full-length chitinase gene, the 5′ and 3′ flanking regions were subjected to thermal asymmetrical interlaced (TAIL)-PCR [31] using Genome Walking Kit (TaKaRa). The final PCR products were agarose gel purified, ligated into a pGEM-T Easy vector and sequenced by Sangon.

Sequence assembly was performed using Vector NTI 10.3 software (InforMax, USA). The signal peptide and functional domains in the deduced amino acid sequence were predicted using SignalP (http://www.cbs.dtu.dk/services/SignalP/) and SMART (http://smart.embl-heidelberg.de/), respectively. DNA sequences were translated to yield corresponding amino acid sequences with ExPASy (http://au.expasy.org). DNA and protein sequence alignments with known chitinase sequences were performed with blastn and blastp (http://www.ncbi.nlm.nih.gov/BLAST/), respectively. The key functional residues were predicted at http://pfam.sanger.ac.uk/search. Multiple sequence alignments were performed with Clustal X [32]. Phylogenetic tree for chitinases was constructed using the neighbor-joining method of MEGA 4.0. One thousand bootstrap repetitions were used to assess the reliability of the tree.

### Expression of ChiCD3 in *E. coli*

The gene fragment encoding ChiCD3 (which lacked the putative signal peptide) was amplified using the genomic *A. veronii* CD3 DNA, *Pfu* Turbo DNA polymerase (TaKaRa), and the primers pET30a-*HindIII* (5′-cccAAGCTTGCCAGGCCGCTTATCCCGCC-3′) and pET30a-*XhoI* (5′-ccgCTCGAGTAAATAGCTACAGGCAGACTT-3′) (*Hind*III and *Xho*I restriction sites are underlined, respectively). The PCR product was purified with TaKaRa Purification Kit, cloned into the *Hind*III and *Xho*I sites of pET-30a(+) and then transformed into *E. coli* BL21(DE3) competent cells. Positive transformants that contained the correct insert were identified by isolating the insert through an agarose gel and sequencing it. These cells were then cultured in 25-ml LB medium containing 100 µM ampicillin at 37°C until the A_600_ of the culture was 0.6–0.8. Protein expression was induced at 15°C by addition of IPTG (final concentration, 1 mM) and the culture was incubated for an additional 12 h.

### Purification and identification of ChiCD3

Cells were centrifuged at 8,000 × *g* for 5 min, washed with sterile 0.7% (w/v) NaCl_(aq)_, suspended in 20 mM Tris-HCl (pH 8.0) and lysed with a microfluidizer (Microfluidics, USA). Soluble and insoluble fractions were separated by centrifugation at 12,000 × *g* for 15 min at 4°C. The (His)_6_-tagged proteins in the supernatant were isolated by Ni^2+^-NTA metal-chelating affinity chromatography using a His-SelectTM cartridge (Sigma) according to the manufacturer's instructions. SDS–polyacrylamide gel electrophoresis (PAGE) used a 12% running gel [33]. The purified enzyme was digested with trypsin and its tryptic peptides were sequenced at the State Key Laboratory of Biology of Biomembrane and Membrane Technology (Institute of Zoology, Chinese Academy of Science) using liquid chromatography-electrospray ionization-tandem mass spectrometry. Protein concentration was determined by the Bradford method [34] with bovine serum albumin as the standard.

### Chitinase activity assay

Chitinase activity was determined based on the amount of *N*-acetylglucosamine (reducing sugar) liberated from colloidal chitin [35]. Reactions containing 500 µl of an enzyme solution and 500 µl of 1% colloidal chitin in 20 mM Tris–HCl (pH 8.0) were incubated at 37°C for 2 h, boiled for 5 min, and cooled to room temperature. The mixtures were centrifuged at 10,000 × *g*, and the amounts of reducing sugars released into the supernatants were determined by the DNS method [36] under standard conditions (pH 6.0, 50°C, 2 h). One unit (U) of chitinase activity was defined as the amount of enzyme required to produce 1 mg of reducing sugar in 2 h.

### Characterization of the physical properties that affect ChiCD3 activity

The effect of pH on enzyme activity was determined at 50°C between pH 3.0 and 10.0. The effect of pH on enzyme stability was determined by measuring the chitinase activity under standard conditions after incubation at 50°C in buffers with different pH values for 2 h. The buffers were McIlvaine buffer (pH 3.0–8.0), 0.1 M Tris–HCl (pH 8.0–9.0), and 0.1 M glycine–NaOH (pH 9.0–12.0). The temperature optimum of the enzyme activity was determined at the optimal pH (pH 6.0) for the temperature range of 20 to 70°C. Thermal stability was determined by measuring the residual activity after incubation at 20°C and 50°C, pH 6.0 for 2 h.

### Substrate specificity and kinetic parameters

The substrate specificity of ChiCD3 was determined by measuring the enzyme activity after incubation in 20 mM Tris–HCl containing 0.5% of each substrate (colloidal chitin, barley β-glucan, locust bean gum, carboxymethyl cellulose and birchwood xylan) at pH 6.0 and 50°C for 2 h. The amount of reducing sugars produced was estimated using DNS method as described above.

To determine the values of *K_m_* and *V_max_* for ChiCD3, the enzyme was incubated with colloidal chitin (0.1% to 1.0%) (w/v) and the amounts of reducing sugars were determined using the DNS method. The values for *K_m_* and *V_max_* were determined from a Lineweaver-Burk plot.

### Degradation of myxospore shell valves by ChiCD3

The shell valve-degrading activities of two commercial chitinases and ChiCD3 were characterized. *S. griseus* chitinase 1 (C6173, Sigma) had a specific activity of ∼200 U g^–1^ against colloidal chitin. *S. marcescens* chitinase 2 (C7809, Sigma) had an activity of 400–1,200 U g^–1^ against colloidal chitin. Aliquots (100 µl) of the myxospore suspension (∼500 mg wet weight in spores) were treated with 19 U of chitinase 1, chitinase 2, or ChiCD3 in 20 mM Tris–HCl (pH 8.0) or with buffer only (control). After incubation at 37°C for 2 h, each supernatant was collected by centrifugation at 3,000 × *g*, 4°C for 5 min and subjected to the DNS assay. Each pellet was observed under the scanning electron microscope. As the intact mature spores have smooth shell and without projections, the spores with damaged shell will be counted as the damaged myxospores after treatment with the chitinase. The myxospores treated with 1.9, 19 or 190 U of ChiCD3 were also examined by optical microscope.

### Nucleotide sequence accession numbers

The nucleotide sequences for the *Thelohanellus kitauei* 18S rDNA gene fragment and the chitinase gene *chiCD3* were deposited in GenBank under accession numbers GU350406 and FJ561294, respectively.
